# Commentary: Comparative Analysis of Phylogenetic Assignment of Human and Avian ExPEC and Fecal Commensal *Escherichia coli* Using the (Previous and Revised) Clermont Phylogenetic Typing Methods and its Impact on Avian Pathogenic *Escherichia coli* (APEC) Classification

**DOI:** 10.3389/fmicb.2017.01904

**Published:** 2017-10-12

**Authors:** Marjanca Starčič Erjavec, Luka Predojević, Darja Žgur-Bertok

**Affiliations:** Molecular Genetics Group, Department of Biology, Biotechnical Faculty, University of Ljubljana, Ljubljana, Slovenia

**Keywords:** *Escherichia coli*, phylogroup, avian, human, phylogenetic typing, fecal *E. coli* (FEC), extraintestinal pathogenic *E. coli* (ExPEC)

*Escherichia coli* (*E. coli*) is mostly a commensal bacterium, part of the intestinal microbiota of a variety of animals, including humans (Bélanger et al., 2011; Vila et al., 2016). However, some *E. coli* strains can be pathogenic and depending on the spectrum of encoded virulence factors *E. coli* can cause either intestinal or extraintestinal infections (Kaper et al., 2004). It is known that the species *E. coli* has an extensive genetic substructure (Chaudhuri and Henderson, 2012) and that the substructure of *E. coli* populations differs among distinct geographical regions (Freitag et al., 2005; Walk et al., 2009) and bacterial hosts (Vadnov et al., 2017).

Initially, four different phylogenetic groups of *E. coli* were defined, A, B1, B2, and D (Chaudhuri and Henderson, 2012). Clermont et al. (2000) established a PCR-method, the so called triplex PCR, for assigning *E. coli* strains into these four phylogenetic groups, a method that was widely used to type and subtype commensal and pathogenic *E. coli*. Phylogenetic classification has been extensively used to compare with serogroup, virulence and resistance traits as well as distribution among various hosts. However, subsequently, on the basis of multi-locus sequence typing and complete genome data, additional *E. coli* phylogenetic groups were recognized (Walk et al., 2009; Luo et al., 2011). The number of defined phylogenetic groups thus rose to 8 (A, B1, B2, C, D, E, F that belong to *E. coli sensu stricto*, and the eighth—the *Escherichia* cryptic clade I). Thus, Clermont et al. (2013) proposed a revised, so called extended quadruplex method for assigning *E. coli* strains to phylogenetic groups that is now replacing the triplex method. The authors validated the extended quadruplex method on a set of 234 strains, which included the ECOR strains (Clermont et al., 2011) and 133 strains from Australia (Gordon et al., 2008). In addition, Clermont et al. (2013) used the new extended quadruplex method for phylogroup assignment of 293 human fecal *E. coli* strains from France and 373 human fecal *E. coli* strains from Australia (Clermont et al., 2013). The authors reported that 12.8% of the tested strains belonged to the new phylogroups C, E, F, and clade I and that strains previously assigned, with the triplex method, to the A and D group should be retested with the new extended quadruplex method. None of the investigated strains were not typeable (NT). Recently, Logue et al. (2017) performed a comparative analysis of phylogenetic assignment of human and avian extraintestinal pathogenic (ExPEC) and fecal commensal *E. coli* (FEC) strains and showed that in total 13.05% of studied human *E. coli* strains and 40.49% of avian *E. coli* strains had to be reclassified. The majority of reassignments among the human *E. coli* strains involved changes from phylogroup D to F (45 out of 139 reclassifications), A to C (29 out of 139 reclassifications) and D to B2 (26 out of 139 reclassifications), while among the avian *E. coli* strains, the majority were reclassified from phylogroup A to C (162 out of 377 reclassifications), D to F (139 out of 377 reclassifications), and D to E (26 out of 377 reclassifications) (Logue et al., 2017). Here, we compared phylogroup classification of our strain collections: *E. coli* from skin and soft tissue infections (Petkovšek et al., 2009), fecal *E. coli* strains from healthy humans (Starčič Erjavec et al., 2010), and avian fecal strains (Salmič and Stele, 2012), with both PCR methods and with the results presented in Logue et al. (2017) (Table 1). Compared to the latter study (Logue et al., 2017), among our strain collections, more human (27.60% of human) and less avian (23.33% of avian) strains had to be reclassified. Further, among our human strains, the majority involved reclassification from the D to E phylogroup (19 out of 53 reclassifications), and D to F (9 out of 53 reclassifications). On the other hand, only 4 out of 53 involved reclassification from A to C as well as from B1 to E, with the latter not reported by Logue et al. (2017). Further, among our avian fecal strains, the majority of reclassifications were from the D to NT (7 out of 21 reclassifications), D to E (5 out of 21 reclassifications) and A to NT (3 out of 21 reclassifications). Our results thus showed that among distinct *E. coli* populations, reclassifications to different groups occurred with different prevalences. This is also evident from our data obtained on our collection of 86 fecal *E. coli* strains from brown bears (Vadnov et al., 2017), where the most prevalent reclassification was from group D to Clade III/IV/V with 25 out of 61 reclassifications, followed by reclassification from D to E (10 out of 61 reclassifications) (Table 1). Further, the high number of reclassifications to NT observed among our avian fecal strains is striking, especially as Logue et al. (2017) reported only a small number for reclassifications to the NT, in total from A to NT only 5 out of 516 reclassifications, while from B1 to NT and from B2 to NT only 1 out of 516 reclassifications. A survey on published studies using the extended quadruplex Clermont method for assigning the phylogenetic group showed even higher numbers of NT *E. coli* strains: 16 *E. coli* strains (7.92%) from 202 *E. coli* strains isolated from in- and outpatients in Burkina Faso (Ouedraogo et al., 2016), 8 *E. coli* strains (24.24%) from 33 vaginal *E. coli* strains isolated from pregnant women in Mozambique (Sáez-López et al., 2016a), 9 *E. coli* strains (6.21%) from 145 vaginal and obstetric infection *E. coli* strains from women in Barcelona (Sáez-López et al., 2016b), 12 *E. coli* strains (3.05%) from 393 *E. coli* strains from mallard ducks in Germany (Rödiger et al., 2015), 38 *E. coli* strains (27.14%) from 140 uropathogenic *E. coli* strains in Iran (Iranpour et al., 2015).

**Table 1:** Changes in phylogenetic group assignment based on application of the revised extended quadruplex Clermont method to assignment as determined by the original triplex Clermont method [our data in black, data from Logue et al. (2017) in red].

***Escherichia coli* from**	**Human SSTI**	**Human ExPEC**	**Human FEC**	**Human FEC**	**Human total**	**Human total**	**Avian FEC**	**Avian FEC**	**Avian total**	**Human and avian total**	**Human and avian total**	**Brown bear FEC**
**Numbers of strains**	**102**	**868**	**90**	**197**	**192**	**1065**	**90**	**199**	**931**	**282**	**1996**	**86**
**Reclassification**	***N*** **(%)**	***N*** **(%)**	***N*** **(%)**	***N*** **(%)**	***N*** **(%)**	***N*** **(%)**	***N*** **(%)**	***N*** **(%)**	***N*** **(%)**	***N*** **(%)**	***N*** **(%)**	***N*** **(%)**
A 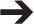 B1		2 (0.23)				2 (0.19)		3 (1.51)	4 (0.43)		6 (0.30)	3 (3.49)
A 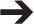 B2	1 (0.98)				1 (0.52)					1 (0.35)		
A 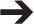 C	3 (2.94)	27 (3.11)	1 (1.11)	2 (1.02)	4 (2.08)	29 (2.72)	1 (1.11)	13 (6.53)	162 (17.40)	5 (1.77)	191 (9.57)	
A 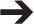 D			2 (2.22)	2 (1.02)	2 (1.04)	2 (0.19)				2 (0.71)	2 (0.10)	
A 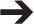 E			2 (2.22)		2 (1.04)		1 (1.11)			2 (0.71)		1 (1.16)
A 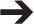 Clade I								3 (1.51)	5 (0.54)		5 (0.25)	
A 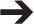 NT		2 (0.23)	3 (3.33)	1 (0.51)	3 (1.56)	3 (0.28)	3 (3.33)		2 (0.21)	6 (2.13)	5 (0.25)	
B1 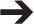 A									1 (0.11)		1 (0.05)	
B1 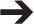 B2				1 (0.51)		1 (0.09)					1 (0.05)	
B1 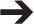 D								2 (1.01)	2 (0.21)		2 (0.10)	1 (1.16)
B1 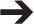 E	1 (0.98)		3 (3.33)		4 (2.08)					4 (1.42)		
B1 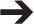 NT		1 (0.12)				1 (0.09)					1 (0.05)	
B2 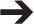 A	2 (1.96)				2 (1.04)		1 (1.11)			3 (1.06)		2 (2.33)
B2 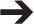 B1									3 (0.32)		3 (0.15)	
B2 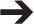 D		4 (0.46)		1 (0.51)		5 (0.47)		3 (1.51)	4 (0.43)		9 (0.45)	
B2 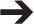 E	1 (0.98)	1 (0.12)	1 (1.11)		2 (1.04)	1 (0.09)	1 (1.11)	5 (2.51)	14 (1.50)	3 (1.06)	15 (0.75)	2 (2.33)
B2 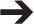 F		2 (0.23)				2 (0.19)		2 (1.01)	5 (0.54)		7 (0.35)	
B2 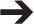 Clade I								1 (0.50)	1 (0.11)		1 (0.05)	
B2 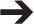 NT		5 (0.58)				5 (0.47)	1 (1.11)			1 (0.35)	5 (0.25)	
D 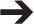 A										3 (1.06)		2 (2.33)
D 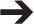 B1		1 (0.12)				1 (0.09)		1 (0.50)	2 (0.21)		3 (0.15)	5 (5.81)
D 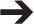 B2	1 (0.98)	22 (2.53)	1 (1.11)	4 (2.03)	2 (1.04)	26 (2.44)			5 (0.54)	2 (0.71)	31 (1.55)	4 (4.65)
D 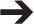 C									2 (0.21)		2 (0.10)	
D 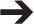 E	6 (5.88)	10 (1.15)	13 (14.44)	1 (0.51)	19 (9.90)	11 (1.03)	5 (5.56)	4 (2.01)	26 (2.79)	24 (8.51)	37 (1.85)	10 (11.63)
D 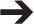 F	3 (2.94)	40 (4.61)	6 (6.67)	5 (2.54)	9 (4.69)	45 (4.23)	1 (1.11)	6 (3.02)	139 (14.93)	10 (3.55)	184 (9.22)	5 (5.81)
D 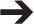 I/II												1 (1.16)
D 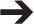 III/IV/V			2 (2.22)		2 (1.04)					2 (0.71)		25 (29.07)
D 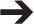 NT	1 (0.98)				1 (0.52)		7 (7.78)			8 (2.84)		
NT 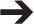 A		1 (0.12)				1 (0.09)					1 (0.05)	
NT 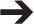 B2		4 (0.46)				4 (0.38)					4 (0.20)	
TOTAL	19 (18.63)	122 (14.06)	34 (37.78)	17 (8.63)	53 (27.60)	139 (13.05)	21 (23.33)	43 (21.61)	377 (40.49)	74 (26.24)	516 (25.85)	61 (70.93)

*NT, not typeable*.

To conclude, Logue et al. (2017) stated that the new extended quadruplex method had a significant impact on avian pathogenic *E. coli* classification and definition of some human uropathogenic strains, a statement we fully support. However, we would like to emphasize that the extent of reclassifications into different groups differ among distinct *E. coli* populations and that, as with the new extended quadruplex method many strains are NT, the question arises whether there is a need for a revised revised phylogenetic typing method? It is well known that *E. coli* is a highly diverse bacterial species therefore, it is not unexpected that all strains from novel environments were not phylotyped with the new extended quadruplex method. We believe that there is a clear need to search for NT *E. coli* strains from novel environments (new hosts in not yet explored geographic regions). Whole genome sequence analysis of such strains should be performed in the search for markers that can be incorporated into a new rapid PCR phylotyping method.

## Author contributions

All authors listed, have made substantial, direct and intellectual contribution to the work, and approved it for publication. MSE led the submission process.

### Conflict of interest statement

The authors declare that the research was conducted in the absence of any commercial or financial relationships that could be construed as a potential conflict of interest.

## References

[B1] Bélanger L. Garenaux A. Harel J. Boulianne M. Nadeau E. Dozois C. M. (2011). Escherichia coli from animal reservoirs as a potential source of human extraintestinal pathogenic E. coli. FEMS Immunol. Med. Microbiol. 62, 1–10. 10.1111/j.1574-695X.2011.00797.x 21362060

[B2] Chaudhuri R. R. Henderson I. R. (2012). The evolution of the *Escherichia coli* phylogeny. Infect. Genet. Evol. 12, 214–226. 10.1016/j.meegid.2012.01.005 22266241

[B3] Clermont O. Bonacorsi S. Bingen E. (2000). Rapid and simple determination of the *Escherichia coli* phylogenetic group. Appl. Environ. Microbiol. 66, 4555–4558. 10.1128/AEM.66.10.4555-4558.2000 11010916 PMC92342

[B4] Clermont O. Christenson J. K. Denamur E. Gordon D. M. (2013). The Clermont *Escherichia coli* phylo-typing method revisited: improvement of specificity and detection of new phylo-groups. Environ. Microbiol. Rep. 5, 58–65. 10.1111/1758-2229.12019 23757131

[B5] Clermont O. Olier M. Hoede C. Diancourt L. Brisse S. Keroudean M.. (2011). Animal and human pathogenic *Escherichia coli* strains share common genetic backgrounds. Infect. Genet. Evol. 11, 654–662. 10.1016/j.meegid.2011.02.005 21324381

[B6] Freitag T. Squires R. A. Schmid J. Elliott J. (2005). Feline uropathogenic *Escherichia coli* from Great Britain and New Zealand have dissimilar virulence factor genotypes. Vet. Microbiol. 106, 79–86. 10.1016/j.vetmic.2004.11.014 15737476

[B7] Gordon D. M. Clermont O. Tolley H. Denamur E. (2008). Assigning *Escherichia coli* strains to phylogenetic groups: multi-locus sequence typing vs. the PCR triplex method. Environ. Microbiol. 10, 2484–2496. 10.1111/j.1462-2920.2008.01669.x 18518895

[B8] Iranpour D. Hassanpour M. Ansari H. Tajbakhsh S. Khamisipour G. Najafi A. (2015). Phylogenetic groups of *Escherichia coli* strains from patients with urinary tract infection in Iran based on the new Clermont phylotyping method. Biomed. Res. Int. 2015:846219. 10.1155/2015/846219 25692147 PMC4322292

[B9] Kaper J. B. Nataro J. P. Mobley H. L. (2004). Pathogenic *Escherichia coli*. Nat. Rev. Microbiol. 2, 123–140. 10.1038/nrmicro818 15040260

[B10] Logue C. M. Wannemuehler Y. Nicholson B. A. Doetkott C. Barbieri N. L. Nolan L. K. (2017). Comparative analysis of phylogenetic assignment of human and avian ExPEC and fecal commensal *Escherichia coli* using the (previous and revised) Clermont phylogenetic typing methods and its impact on avian pathogenic *Escherichia coli* (APEC) classification. Front. Microbiol. 8:283. 10.3389/fmicb.2017.00283 28280491 PMC5322314

[B11] Luo C. Walk S. T. Gordon D. M. Feldgarden M. Tiedje J. M. Konstantinidis K. T. (2011). Genome sequencing of environmental *Escherichia coli* expands understanding of the ecology and speciation of the model bacterial species. Proc. Natl. Acad. Sci. U.S.A. 108, 7200–7205. 10.1073/pnas.1015622108 21482770 PMC3084108

[B12] Ouedraogo A. S. Sanou M. Kissou A. Sanou S. Solaré H. Kaboré F.. (2016). High prevalence of extended-spectrum ß-lactamase producing *enterobacteriaceae* among clinical isolates in Burkina Faso. BMC Infect. Dis. 16:326. 10.1186/s12879-016-1655-3 27400864 PMC4939587

[B13] Petkovšek Ž. Eleršič K. Gubina M. Žgur-Bertok D. Starčič Erjavec M. (2009). Virulence potential of *Escherichia coli* isolates from skin and soft tissue infections. J. Clin. Microbiol. 47, 1811–1817. 10.1128/JCM.01421-08 19357208 PMC2691073

[B14] Rödiger S. Kramer T. Frömmel U. Weinreich J. Roggenbuck D. Guenther S.. (2015). Intestinal *Escherichia coli* colonization in a mallard duck population over four consecutive winter seasons. Environ. Microbiol. 17, 3352–3361. 10.1111/1462-2920.12807 25684458

[B15] Sáez-López E. Cossa A. Benmessaoud R. Madrid L. Moraleda C. Villanueva S.. (2016a). Characterization of vaginal *Escherichia coli* isolated from pregnant women in two different African sites. PLoS ONE 11:e0158695. 10.1371/journal.pone.0158695 27387665 PMC4936694

[B16] Sáez-López E. Guiral E. Fernández-Orth D. Villanueva S. Goncé A. López M.. (2016b). Vaginal vs. obstetric infection *Escherichia coli* isolates among pregnant women: antimicrobial resistance and genetic virulence profile. PLoS ONE 11:e0146531. 10.1371/journal.pone.0146531 26784330 PMC4718642

[B17] Salmič J. Stele F. (2012). Filogenetska analiza ptičjih izolatov bakterije Escherichia coli z metodo verižne reakcije s polimerazo: Raziskovalna naloga [Phylogenetic Analysis of Escherichia coli Avian Isolates with the Polymerase Chain Reaction: Research Project]. Ljubljana: Gimnazija Poljane.

[B18] Starčič Erjavec M. Jesenko B. Petkovšek Ž. Žgur-Bertok D. (2010). Prevalence and associations of *tcpC*, a gene encoding a toll/interleukin-1 receptor domain-containing protein, among *Escherichia coli* urinary tract infection, skin and soft tissue infection, and commensal isolates. J. Clin. Microbiol. 48, 966–968. 10.1128/JCM.01227-09 20042631 PMC2832412

[B19] Vadnov M. Barbič D. Žgur-Bertok D. Starčič Erjavec M. (2017). *Escherichia coli* isolated from faeces of brown bears (*Ursus arctos*) have a lower prevalence of human extraintestinal pathogenic *E. coli* virulence-associated genes. Can. J. Vet. Res. 81, 59–63. 28154465 PMC5220600

[B20] Vila J. Sáez-López E. Johnson J. R. Römling U. Dobrindt U. Cantón R.. (2016). *Escherichia coli*: an old friend with new tidings. FEMS Microbiol. Rev. 40, 437–463. 10.1093/femsre/fuw005 28201713

[B21] Walk S. T. Alm E. W. Gordon D. M. Ram J. L. Toranzos G. A. Tiedje J. M.. (2009). Cryptic lineages of the genus *Escherichia*. Appl. Environ. Microbiol. 75, 6534–6544. 10.1128/AEM.01262-09 19700542 PMC2765150

